# The Hon. Sir Arthur Stanley's Visit

**Published:** 1918-06-22

**Authors:** 


### NURSING AFFAIRS IN IRELAND.

### The Hon. Sir Arthur Stanley's Visit.

The importance and value of Sir Arthur Stanley's visit to Ireland cannot be over-estimated. It is not too much to say that he carried Dublin by storm. His charm of manner, his broad outlook, his lofty superiority to everything that savoured of pettiness—these qualities, combined with his intense earnestness, have won golden opinions even in camps which had regarded his coming with suspicion, if not with disfavour. During his brief stay, extending over three days, he had not an idle moment, and the feeling that is left behind is one of satisfaction and gladness that the champion of the Red Cross and of the hospital nurse has been brought into personal contact with Irish war work and war workers.

The Tribute to Nurses' Fund.

It was in order to launch the Irish Tribute Fund to Nurses that Sir Arthur Stanley came to Dublin, and so a great public meeting was held on the afternoon of his arrival, the 13th inst., in the lecture theatre of the Royal Dublin Society. A guard of honour, consisting of V.A.D.s, lined the hall, and the spacious building was filled with representative citizens and a large contingent of Dublin hospital nurses. On the platform was one of the most brilliant and distinguished assemblages that Dublin has witnessed since the outbreak of the war, a fact which augurs well for the ultimate success of the movement. These included the nobility, the judges, the leading business men, without distinction of creed or politics, amongst them being the following: The Marchioness of Waterford, the Countess of Fingall, Lady Talbot de Malahide, Lady Arnott, D.B.E., the Countess of Kenmare, Lady de Freyne, the Hon. Mrs. Barry, Lady Magrath, Mrs. Shortt (wife of the Chief Secretary), Lord Justice Molony, the President of the College of Surgeons, Sir Horace Plunkett, Mr. Justice Ross, P.C., Mr. Jonathan Hogg, J.C., Sir Dunbar Barton, Sir John Lumsden, M.D., Sir William and Lady Fry, Sir William Gallagher, Sir John Arnott, Bart. (Chairman of the *Irish Times*), Mr. Wm. Martin Murphy (proprietor of the Irish *Daily Independent*), Sir Thomas Stafford, and others. Sir James Campbell, Bart., Lord Chancellor of Ireland, presided.

The Lord Chancellor, who is an accomplished and popular speaker, in introducing Sir Arthur Stanley, paid a brief tribute to the splendid achievements of Ireland's women in connection with the war, and especially to the work of the nurses. Concluding, Sir James Campbell said that it was proposed to raise a comparatively large sum—£10,000—but he did not think it would be a very difficult task. Sir John Arnott had already contributed £300 and Lady Arnott £100. A good start had thus been made, and he commended the movement to the generosity of the Irish race. By Saturday last the Fund had exceeded £1,000.

Sir Arthur Stanley's Speech.

The Hon. Sir Arthur Stanley, who was received with loud applause, said that he felt it an honour to address such an assembly, and he was very pleased that so many had thought it worth while to come there, forsaking the pleasures of the few fine days of the year outside. It had been said in criticism of the Fund they were trying to raise that this was not the moment to make the appeal, and that other funds were more urgently needed. He had read an account from their Commissioner in France of the tragedy of the bombing of a Red Cross hospital by the Germans, and he then realised once more that the nurses had shown the courage so pre-eminently characteristic of them. They were spared from that particular invasion of the Huns in Ireland, but he had been privileged to witness every single bombing raid on London, and had added a few others, but the account of the raid on the hospital was one of the worst he had ever read. On the first occasion, a week previously, the Germans possibly might not have known that they were really bombing a hospital, but on the second occasion it was made perfectly clear that they knew what they were doing—their aeroplanes flew low, set a hut or two on fire, and set up flares so that the whole place was as light as day, and then proceeded deliberately to bomb the hospitals, in which they knew were the sick, the wounded, and the nurses.

Ireland and the Tribute Fund.

He mentioned that fact simply because the account, which was one of the worst he had read, ended by stating that the nurse who was on duty went through the wards and was killed. He did not think they could have a finer tribute to the nurses than the fact that that woman should have gone firmly through her duty, which led her right into the middle of the whole bombing. These women surely deserved some reward, and could they possibly think of a better reward than that when they came home they should find the people had not been unmindful of them and of their work? Therefore, he thought this was the right moment to make the appeal, and he was enormously encouraged by that meeting, and by the very kindly way this Tribute Fund had been received in Ireland. In connection with the large number of nurses they employed at the present moment—well over 3,000—they found two things—there was a curious lack of organisation of a profession so old and honourable as that of nursing, and there was no fund of anything like adequate dimensions to provide relief for those nurses who were, owing to sickness or any other cause, unable to follow their vocation. The Red Cross had at first set aside a sum of £10,000 to help Red Cross nurses, but that fund had been largely drawn upon. They then learned that a vast number of nurses were working in the Army or the Red Cross, and in the event of their suffering because of their work they would receive compensation from the Government. That, of course, provided for a very large number of cases, but, as they knew, compensation from the Government was not as a rule over-generous, and it was often a very long time in coming. They felt, therefore, that in the case of nurses who would rightly get compensation there was still room for a fund such as that now proposed. There was another class of nurses not engaged in war work, or serving in the Army—a vast army of nurses working for the civil population, and it would be very poor return to the soldier if the home he loved were being neglected. These nurses were receiving no compensation from the Government, and it was very largely for them he was asking their support. Another point to remember was that, owing to the time required for training, very few nurses could really begin to earn any substantial pay until they arrived at the age of about twenty-five years. Nursing was very hard and onerous work, and a large number were not able to follow it to a late period in life. Moreover—and he said it with all respect to the governors of hospitals—the pay of nurses was not adequate to the services they rendered.

The Need for the Fund.

They, therefore, had got to deal with women who began to earn money comparatively late in life, and could not go on working to an old age. Could they possibly be expected to build up a large relief fund to help their less fortunate sisters? When the war was over they would have to deal with a number of nurses requiring rest, good food, and special attention, if they were to remain healthy and recuperate after what they had gone through. What sick and broken-down nurses wanted was immediate help, and that help they were preparing to give. The two objects they had in view were the co-ordination of the nursing profession and the helping of those who had fallen by the way. The first object would be looked after by the College of Nursing, and he need not go into that,

**SIR ARTHUR STANLEY IN IRELAND**—(*cont.*)

because it had nothing to do with the subject they were there to discuss, which had been rightly called the Tribute Fund. It was not charity—there was nothing degrading to the nurse in accepting the gratitude of a whole nation, a tribute from the heart of the great British nation to those who had done so well in the war, and who had thought so much of others that they had no time to give a thought to themselves. The Irish were not only a generous, but a warm-hearted, race, and he was, therefore, certain they would get what they were asking for, and probably a great deal more. He was glad to find also that they were a very hard-headed race, and if they gave money they liked to see how that money was going to be spent, and would like to have the spending of it themselves. Well, if they wanted to do a good thing well, especially in the spending of money, they should do it themselves, and he was bound to say that the request from the Executive Committee to the effect that the money raised in Ireland should be spent through the Irish Committee upon Irish nurses seemed pre-eminently a sensible one. If the Irish Committee could take on themselves the responsibility of saying that they would provide for all Irish nurses then he could assure them of their most deep and sincere gratitude. At the same time, he asked them to allow the headquarters in London, who were raising money for the whole British Empire, to allow them to look upon the money raised in Ireland as part of the nation's fund, and as the contribution from Ireland to the nurses of the whole British Empire, and as he did not see the immediate connection between the two he asked them to allow him (the speaker) as their humble servant to be the connecting link, for the time being, at all events. He would go back to London and tell them that whatever Ireland wished to do would be well done, and he was perfectly confident they would be satisfied with that assurance.

A vote of thanks to Sir Arthur Stanley was proposed by the President of the Royal College of Surgeons (Mr. J. B. Storey). Mr. Storey, in the course of his remarks, mentioned that he had been particularly requested not to attend this meeting. Of course, it is needless to suggest the quarter from which such a request emanated. Sir Horace Plunkett, in seconding, said the more money they got and quickly the more gratifying it would be to their pride in their country. The vote was carried with acclamation.

Sir Arthur Stanley, accompanied by Lord Donoughmore, K.P., Chairman of the Joint V.A.D. for Ireland, and Lieutenant Arnott, A.D.C., inspected various hospitals during the last three days of the week, including the Irish Counties War Hospital, Dublin Castle Hospital, the Duke of Connaught's and Princess Patricia's Hospital at Bray. On Saturday afternoon Sir Arthur Stanley and Lord Donoughmore were entertained to luncheon at the Shelbourne Hotel, Dublin, by Sir John Lumsden, M.D., there being about sixty other guests, and at the conclusion of the function they inspected a muster of over a thousand V.A.D.s, men and women, in Lord Iveagh's beautiful gardens, St. Stephen's Green, and awarded the valuable prizes won in competitions which preceded the parade. During Sir Arthur Stanley's stay in Ireland he was the guest of Lady Arnott, D.B.E., Mountmerrion. IERNE.

